# IFN-**λ** uniquely promotes CD8 T cell immunity against SARS-CoV-2 relative to type I IFN

**DOI:** 10.1172/jci.insight.171830

**Published:** 2024-05-21

**Authors:** Abigail D. Solstad, Parker J. Denz, Adam D. Kenney, Najmus S. Mahfooz, Samuel Speaks, Qiaoke Gong, Richard T. Robinson, Matthew E. Long, Adriana Forero, Jacob S. Yount, Emily A. Hemann

**Affiliations:** 1 Department of Microbial Infection and Immunity, The Ohio State University College of Medicine, Columbus, Ohio, USA.; 2 Infectious Diseases Institute, The Ohio State University, Columbus, Ohio, USA.; 3 Dorothy M. Davis Heart and Lung Research Institute and; 4 Department of Internal Medicine, Division of Pulmonary, Critical Care, and Sleep Medicine, The Ohio State University College of Medicine, Columbus, Ohio, USA.

**Keywords:** Immunology, Infectious disease, Innate immunity, Mouse models, T cells

## Abstract

Optimization of protective immune responses against SARS-CoV-2 remains an urgent worldwide priority. In this regard, type III IFN (IFN-λ) restricts SARS-CoV-2 infection in vitro, and treatment with IFN-λ limits infection, inflammation, and pathogenesis in murine models. Furthermore, IFN-λ has been developed for clinical use to limit COVID-19 severity. However, whether endogenous IFN-λ signaling has an effect on SARS-CoV-2 antiviral immunity and long-term immune protection in vivo is unknown. In this study, we identified a requirement for IFN-λ signaling in promoting viral clearance and protective immune programming in SARS-CoV-2 infection of mice. Expression of both IFN and IFN-stimulated gene (ISG) in the lungs were minimally affected by the absence of IFN-λ signaling and correlated with transient increases in viral titers. We found that IFN-λ supported the generation of protective CD8 T cell responses against SARS-CoV-2 by facilitating accumulation of CD103^+^ DC in lung draining lymph nodes (dLN). IFN-λ signaling specifically in DCs promoted the upregulation of costimulatory molecules and the proliferation of CD8 T cells. Intriguingly, antigen-specific CD8 T cell immunity to SARS-CoV-2 was independent of type I IFN signaling, revealing a nonredundant function of IFN-λ. Overall, these studies demonstrate a critical role for IFN-λ in protective innate and adaptive immunity upon infection with SARS-CoV-2 and suggest that IFN-λ serves as an immune adjuvant to support CD8 T cell immunity.

## Abstract 2

A model of mouse-adapted SARS-CoV-2 reveals that type III interferon signaling uniquely regulates DC function to promote SARS-CoV-2–specific CD8^+^ T cell responses.

## Graphical abstract

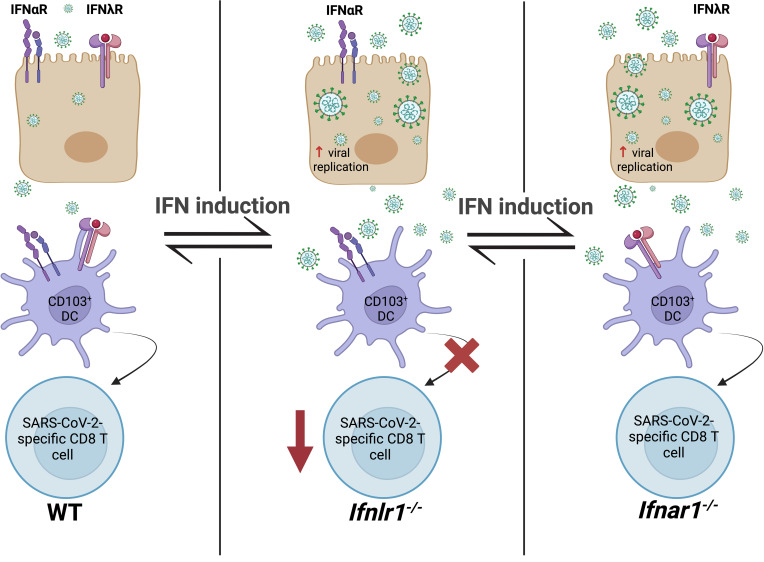

## Introduction

The SARS-CoV-2 pandemic has led to significant global morbidity and mortality, with a devastating economic effect. SARS-CoV-2 infections remain an ongoing public health concern as new variants continue to emerge with increased immune evasion and transmissibility (1–4). Although progress has been made to understand key aspects of COVID-19 pathophysiology, additional insights into the quality and magnitude of immune responses required for virus clearance, tissue repair, and protection against severe disease are needed to drive the development of improved therapeutics and vaccines.

The initial host detection of viral infection by intracellular receptors induces type I and type III IFN secretion, which functions to increase expression of antiviral genes and create a broad antiviral state within tissues (5–7). However, SARS-CoV-2 utilizes strategies that reduce and delay the induction type I and type III IFN responses to evade antiviral immunity (6, 8, 9). Although IFNs are critical for host antiviral defense, hyperactive IFN responses can cause damaging inflammation and tissue pathology (10). Type III IFN (IFN-λ) is produced at mucosal barriers where it mediates antiviral defense through the upregulation of IFN-stimulated genes (ISGs) (5, 7, 11, 12). Compared with type I IFN (IFN-α/β), IFN-λ exerts its antiviral function with less inflammation and tissue damage (13). In the context of respiratory virus infections (e.g., influenza virus and SARS-CoV-2), these properties position IFN-λ as a superior therapeutic compared with type I IFN (13–17) and, in clinical trials, recombinant human IFN-λ was shown to decrease time to clearance and reduce risk of hospitalization during COVID-19 (18–20). However, it is still not well understood whether and how endogenously produced IFN-λ regulates lung pathogenesis or adaptive immune mechanisms following SARS-CoV-2 infection. An increased understanding of IFN-λ on the immune response and lung recovery dynamics will inform the use of IFN-λ–based therapeutic strategies for respiratory infections.

In addition to its antiviral functions, emerging evidence has indicated that IFN-λ regulates cellular immune responses during infection (7, 21). Previously, our work and that of others demonstrated a critical role for IFN-λ in protective responses of adaptive immune cells against influenza virus infection (22, 23). For example, we identified a role for IFN-λ in the generation of influenza virus–specific CD8 T cell responses. In the absence of IFN-λ signaling, mice were more susceptible to heterosubtypic influenza virus rechallenge due to impaired CD8 T cell immunity (22). However, the generality of this unique paradigm and the underlying mechanisms remain unaddressed.

Type I IFN signaling has also been reported to potentiate the CD8 T cell response against influenza virus infection (24), but the contribution of type I IFN to adaptive immunity against betacoronaviruses (e.g., SARS-CoV-2) is less understood. Type I IFN signaling has been examined in human angiotensin-converting enzyme 2–overexpression (ACE2-overexpression) mouse models of SARS-CoV-2 infection and was shown to contribute to development of a robust T cell response, but the relevance of this model for studying immune mechanisms and pathogenesis is unclear due to the overexpression of the viral receptor, which allows nearly all lung cell types to become infected and to produce viral antigens (25). Whether type I IFN influences T cell responses when SARS-CoV-2 infection occurs via native expression of ACE2 has not yet been examined.

Importantly, SARS-CoV-2 is known to elicit CD8 T cell responses following infection (26–28), and robust CD8 responses to either infection or vaccination positively correlate with protection from severe COVID-19. Furthermore, a significant subset of SARS-CoV-2–specific CD8 T cells are cross-reactive against emerging variants of concern (VOC) (28–32), suggesting that they may provide protection in situations where previously existing neutralizing antibodies are evaded. Understanding whether type I and type III IFN regulate CD8 T cell responses is needed and will reveal overlapping or distinct functions of these cytokines.

Here, we report that mice lacking the IFN-λ receptor (*Ifnlr1^–/–^*) experience exacerbated disease, including increased weight loss and virus burden, when infected with mouse-adapted SARS-CoV-2 (strain MA10) (33). Intriguingly, we observed modest temporal changes in the expression of certain ISGs but observed no overt changes in type I or III IFN. This correlated with an increase in virus titers in *Ifnlr1^–/–^* early in infection compared with WT. Our cellular analysis of the immune response revealed an increase in CD8 T cell–priming CD103^+^ DC in the lungs of *Ifnlr1^–/–^* mice compared with WT on day 4 after infection (p.i.). Indeed, SARS-CoV-2–specific CD8 T cells in the lungs of *Ifnlr1^–/–^* mice were reduced at an effector time point (day 8 p.i.). This diminished virus-specific CD8 T cell response was apparent in lung draining lymph nodes (dLN) of *Ifnlr1^–/–^* on day 4 p.i. and accompanied by a decrease of CD103^+^ DC in dLN where they provide T cell activation. In addition, SARS-CoV-2–specific CD8 T cell populations were also reduced in dLN on day 4 p.i. of *Ifnlr1^–/–^* mice compared with WT. In vitro coculture experiments revealed that IFN-λ signaling is required specifically in DC for upregulation of costimulatory molecule expression and activation of CD8 T cells. Strikingly, the reduction in SARS-CoV-2–specific CD8 T cell immunity is specific to *Ifnlr1^–/–^* mice and was not observed in mice lacking the type I IFN receptor (*Ifnar1^–/–^*). Overall, these data reveal a critical and nonredundant role for IFN-λ in driving DC functionality and adaptive immune CD8 T cell responses against SARS-CoV-2.

## Results

### IFN-λ restricts SARS-CoV-2 replication in vivo.

**Figure 1:**
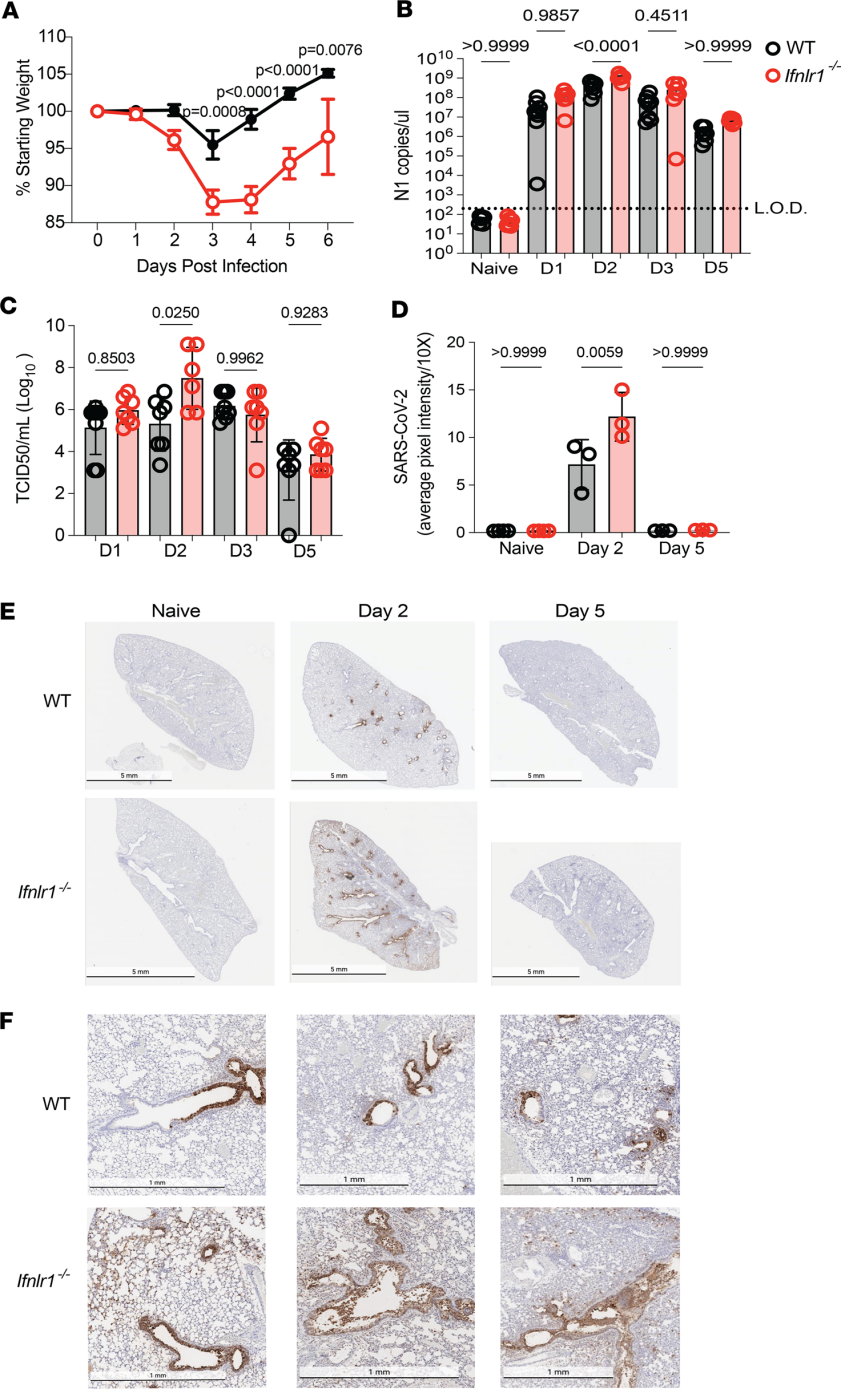
IFN-λ signaling restricts virus replication in a murine model of SARS-CoV-2 infection. (**A**) WT and *Ifnlr1^–/–^* mice were infected intranasally with 1 × 10^5^ TCID_50_ of SARS-CoV-2 MA10, and weight loss was monitored for 6 days. A 2-way ANOVA with Šidák’s multiple-comparison test determined significance. Data from 2 independent experiments pooled with data representing mean ± SEM. *n* = 10 mice/group. (**B**) At days 1, 2, 3, and 5 p.i., RNA was isolated from lungs of naive or infected mice. RNA was subjected to qPCR to determine N1 copies per μl. Data from 2 independent experiments pooled with data representing mean ± SEM. *n* = 6-8 mice/group. (**C**) Lungs were harvested from naive and infected mice at days 1, 2, 3, and 5 p.i., and virus was quantified by TCID_50_. Data from 2 independent experiments pooled with data representing mean ± SEM. *n* = 6–8 mice/group. Statistical significance in **B** and **C** was determined by 1-way ANOVA with Tukey’s multiple-comparison test. (**D**) ImageJ was utilized to quantify the average pixel intensity of 10 randomly selected 10× images from each lung. Significance was determined by 1-way ANOVA with Tukey’s multiple-comparison test, *n* = 3 mice/group with data representing mean ± SD. (**E**) IHC was performed to detect the SARS-CoV-2 nucleocapsid (N). Scale bar: 5 mm. (**F**) Representative images of SARS-CoV-2 N staining from individual animals on day 2 p.i. Scale bar: 1 mm.

To assess the effects of IFN-λ on SARS-CoV-2 replication and pathogenesis, we infected WT C57BL/6J mice and those lacking the IFN-λ receptor (*Ifnlr1^–/–^*) with mouse adapted SARS-CoV-2 (strain MA10) (16, 33). *Ifnlr1^–/–^* mice showed increased weight loss starting on day 2 p.i. and a delayed time to recovery as compared with WT animals (Figure 1A). Pulmonary virus loads were significantly increased in *Ifnlr1^–/–^* mice on day 2 p.i. as indicated by measurement of viral nucleocapsid RNA expression (Figure 1B), infectious virus titer (Figure 1C), and SARS-CoV-2 antigen staining in lung sections (Figure 1D). These results demonstrate that IFN-λ limits SARS-CoV-2–induced weight loss and virus burden, consistent with other recently published studies (17, 34). Histological analysis revealed that SARS-CoV-2 antigens were largely retained in the airways of WT lungs (Figure 1, E and F). Strikingly, in the absence of IFN-λ signaling, dissemination of viral antigen throughout lung sections was apparent compared with WT on day 2 p.i. (Figure 1E). Furthermore, we observed full involvement of airways in *Ifnlr1^–/–^* lungs compared with partial airway infection of WT lungs (Figure 1F). Together, these results demonstrate a previously unreported contribution of IFN-λ in limiting the spread of SARS-CoV-2 viral antigen in the airways and throughout lung tissue that associates with beneficial infection outcomes.

### IFN-λ alters the magnitude of lung transcriptional responses to SARS-CoV-2 infection.

**Figure 2:**
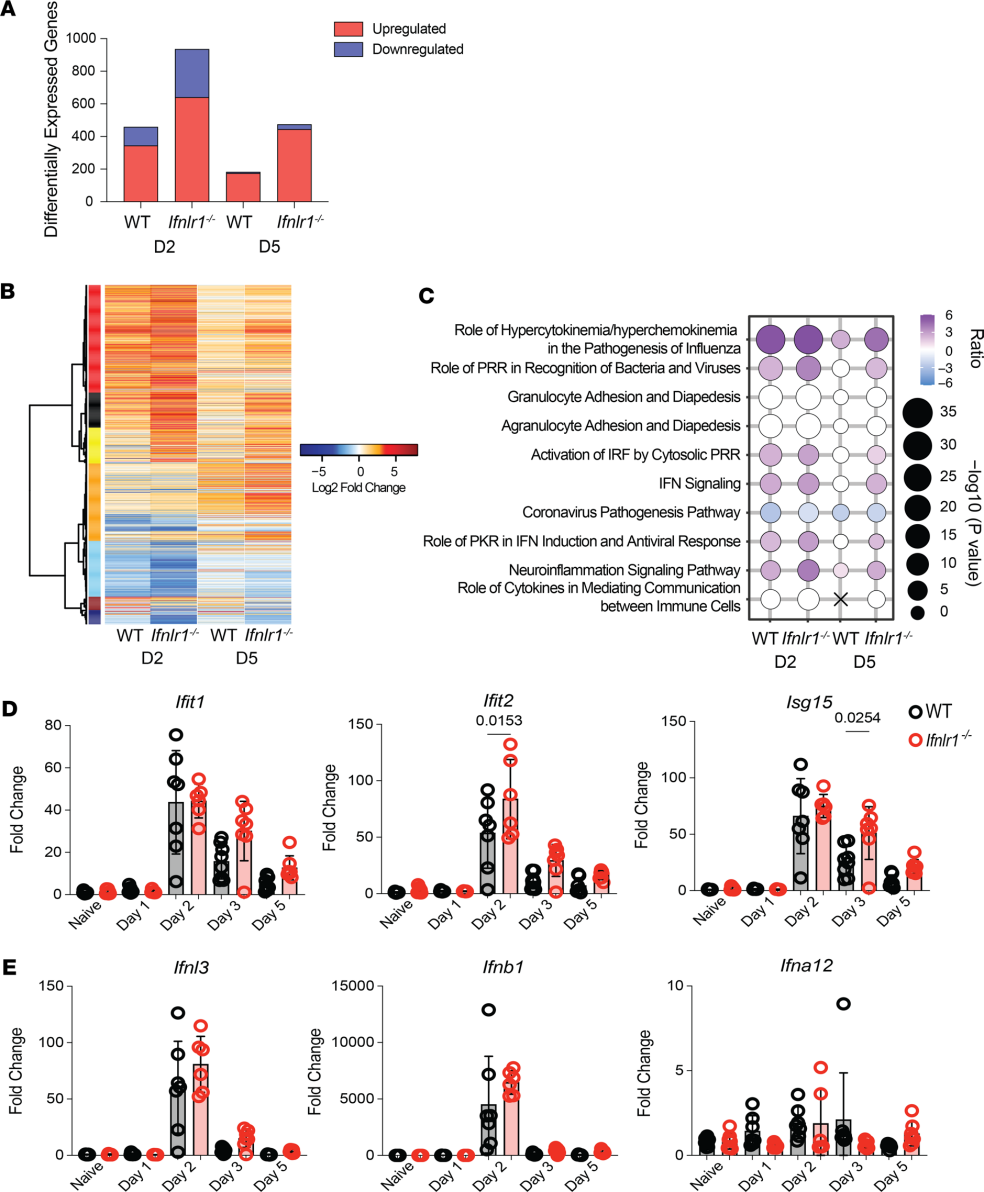
IFN-λ signaling regulates pulmonary transcriptional programming during SARS-CoV-2 MA10 infection. RNA-Seq was performed on whole lungs of WT and *Ifnlr1^–/–^* naive mice or on days 2 and 5 following SARS-CoV-2 infection. (**A**) Quantification of differentially expressed genes (DEG) across WT or *Ifnlr1^–/–^* on days 2 and 5 following infection. Bar graphs represent total number of genes upregulated (red) or downregulated (blue) at the indicated time points. (**B**) Hierarchical clustering of the union of significantly up- or downregulated genes in WT or *Ifnlr1^–/–^* on days 2 and 5 following infection relative to naive. Heatmap represents the log_2_ fold change expression of 1277 DEG. Biweight midcorrelation was used to cluster transcripts in coexpression modules indicated by colors. (**C**) Enrichment of IPA canonical pathways from gene signatures derived from WT or *Ifnlr1^–/–^* mice during SARS-CoV-2 MA10 infection. Bubble plot represents the top 10 significantly enriched canonical pathways across day 2 and day 5 p.i. Bubble size indicates the -log_10_ *P* value of enrichment. Color indicates the inferred pathway activation *Z* score. Color indicates directionality of activation (purple) or repression (blue). White bubbles indicate significant pathways with no inferred activation state. (**D** and **E**) WT and *Ifnlr1^–/–^* mice were infected intranasally with 1 × 10^5^ TCID_50_ of SARS-CoV-2 MA10, and RNA was isolated from lungs of naive or infected mice on days 1, 2, 3, and 5 p.i. (**D**) Relative expression of *Ifit1*, *Ifit2,* and *Isg15* compared with the housekeeping gene *Chmp2a* was determined by qPCR. (**E**) Relative expression of *Ifnl3*, *Ifnb1*, and *Ifna12* compared with the housekeeping gene *Chmp2a* was determined by qPCR. Statistical significance was calculated by 1-way ANOVA followed by Tukey’s multiple-comparison test. Two independent experiments pooled with data representing mean ± SEM. *n* = 6–11 mice/group.

IFN-λ plays an important antiviral role in regulating SARS-CoV-2 replication in vitro through upregulation of ISGs (35–37). Additionally, treatment of mice with recombinant IFN-λ prior to or following SARS-CoV-2 challenge leads to an increase in ISG expression (17, 34). However, the dependence of ISG responses on endogenous IFN-λ signaling during SARS-CoV-2 infection in vivo has not yet been described. To better understand how endogenous type III IFN signaling confers protection against SARS-CoV-2 challenge, we performed global transcriptomic analysis on lungs of WT and *Ifnlr1^–/–^* mice following infection (Supplemental Figure 1D; supplemental material available online with this article; https://doi.org/10.1172/jci.insight.171830DS1). Statistical analysis of differential gene expression (log_2_ fold change, |2|; adjusted *P* < 0.01) demonstrated that SARS-CoV-2 infection elicited a higher number of differentially expressed genes (DEG) in *Ifnlr1^–/–^* relative to WT mice, both at days 2 and 5 p.i. (Figure 2A). Hierarchical clustering of the DEG demonstrated that the loss of *Ifnlr1* expression led to a greater change in the magnitude of DEG (Figure 2B). Functional enrichment analysis of transcriptional profiles revealed that there was a robust enrichment of antiviral and IFN signaling pathways in both WT and *Ifnlr1^–/–^* mice. The increase in the loss of IFN-λ signaling led to a significant increase (–log_10_ *P* value) in the activation ratio of transcripts involved in innate immune antiviral responses (Figure 2C). We validated these inferences by measuring the expression of ISG from whole lungs across independent data sets. Consistent with our RNA-Seq data, we found a significant and transient increase in the early expression of a subset of ISGs (*Ifit2* on day 2 p.i. and *Isg15* on day 3 p.i.), while the expression levels of *Ifit1* remained unchanged (Figure 2D). The differences in ISG expression were not dependent on changes in IFN gene expression, as *Ifnl3*, *Ifnb1*, and *Ifna12* were not vastly altered throughout the course of infection (days 1, 2, 3, or 5 p.i.) between WT and *Ifnlr1^–/–^* mice (Figure 2E). Together with our observations in Figure 1, these data indicate that IFN-λ signaling is necessary to limit local virus spread and early viral replication (day 2 p.i.). The early increases in viral antigen in turn likely lead to increases in type I IFN–dependent ISG expression. These data demonstrate that the unique and nonredundant roles for type I and type III IFN in conferring early protection against respiratory pathogens (38–40) is conserved during SARS-CoV-2 infection.

### IFN-λ signaling alters Cxcl10 and Il10 without affecting Cxcl1, Il6, or neutrophil infiltration during SARS-CoV-2 infection.

**Figure 3:**
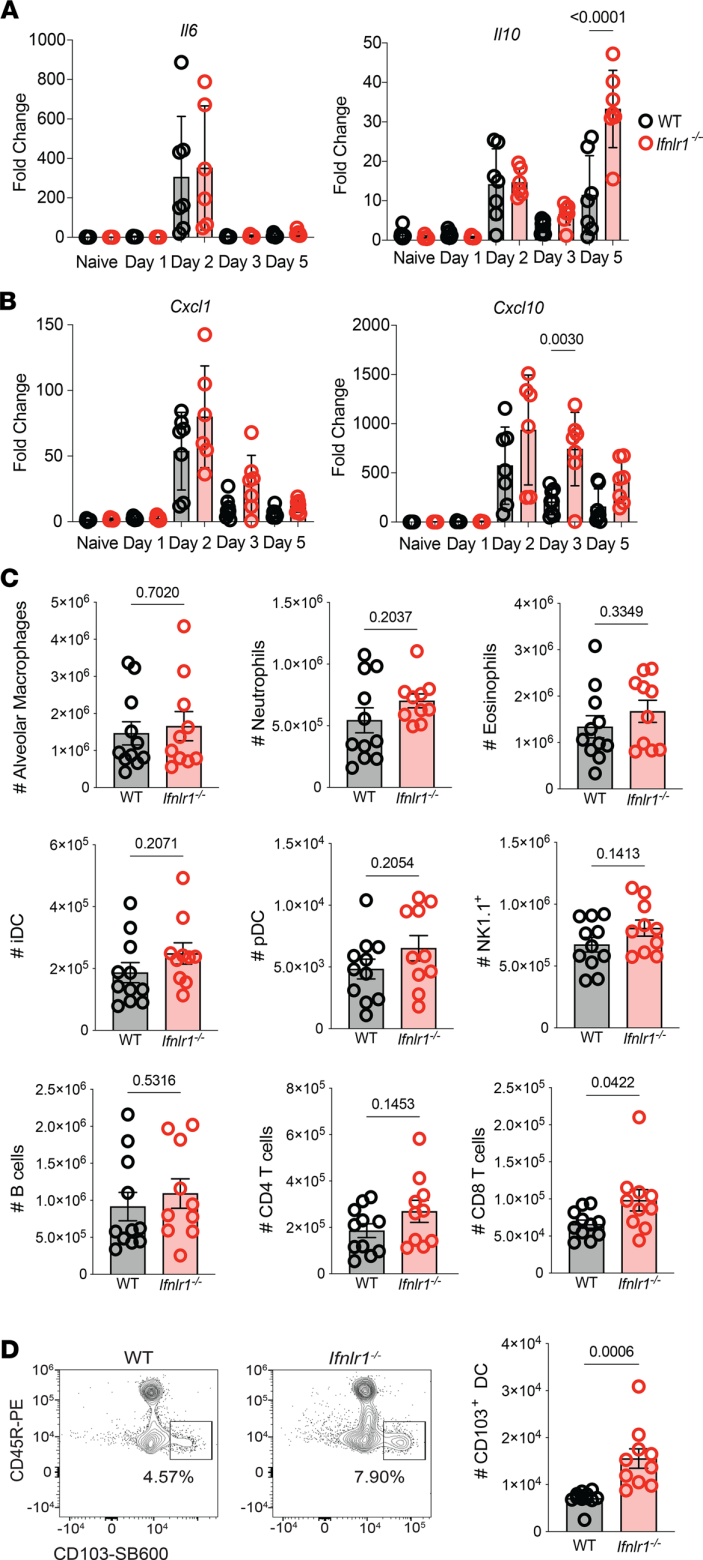
IFN-λ signaling regulates CD103^+^ DC populations in the lungs. (**A** and **B**) WT and *Ifnlr1^–/–^* mice were infected intranasally with 1 × 10^5^ TCID_50_ of SARS-CoV-2 MA10, and RNA was isolated from lungs of naive or infected mice on days 1, 2, 3, and 5 p.i. Relative expression of *Il6* and *Il10* (**A**), as well as Cxcl1 and *Cxcl10* (**B**), compared with the housekeeping gene *Chmp2a* was determined by qPCR. Statistical significance was calculated by 1-way ANOVA followed by Tukey’s multiple-comparison test. Two independent experiments pooled with data representing mean ± SEM. *n* = 6–11 mice/group. (**C** and **D**) WT and *Ifnlr1^–/–^* mice were infected intranasally with 1 × 10^5^ TCID_50_ of SARS-CoV-2 MA10. On day 4 p.i., lungs were harvested and numbers of specific immune cell (CD45^+^) populations were determined by flow cytometry. (**C**) Total numbers of alveolar macrophages, neutrophils, eosinophils, CD11c^+^CD11b^+^MHCII^+^ cells (iDC), pDCs, NK cells (NK1.1+), B cells, CD4 T cells, and CD8 T cells in the lungs was and N_219_-specific CD8 T cells were determined by flow cytometry. (**D**) Representative flow plots displaying the frequency and graphs quantifying numbers of WT and *Ifnlr1^–/–^* mice at day 4 following SARS-CoV-2 MA10 infection. Data from 2 independent experiments pooled with data representing mean ± SEM. *n* = 10–11 mice/group. Statistical significance was determined by unpaired 2-tailed *t* test. Frequencies on representative flow plots represent the percentage of parent gate (directly upstream of gate named in figure) with gating strategy shown in Supplemental Figure 2.

Excessive inflammation associated with increased IL-6 responses and neutrophil infiltration have been implicated in enhanced COVID-19 disease following SARS-CoV-2 infection of humans (41). IFN-λ signaling has been shown to dampen inflammatory responses through modulating neutrophil function (42–45). To determine whether the increased morbidity in *Ifnlr1^–/–^* was associated with increased IL-6 responses, we examined changes in the mRNA expression levels of *Il6*. No statistically difference in expression was observed between WT and *Ifnlr1^–/–^* infected lungs on days 1, 2, 3, and 5 p.i. (Figure 3A). We then examined whether IFN-λ signaling was necessary to mediate neutrophil infiltration and function. We evaluated the expression of *Cxcl1* and *Cxcl10* expression and found no statistically significant difference in *Cxcl1*, while *Cxcl10* was significantly increased only on day 3 p.i. in *Ifnlr1^–/–^* compared with WT (Figure 3B), suggesting that there may be alterations in infiltration of immune cells to the lungs around the time of this increased expression.

We then measured the lung expression of Gr1, a marker expressed in neutrophils and myeloid cells (46), by IHC staining (Supplemental Figure 1B). We did not observe statistical differences in Gr1 positivity between WT and *Ifnlr1^–/–^* on days 2 and 5 p.i. (Supplemental Figure 1B). Additionally, levels of neutrophil elastase, which is released by activated neutrophils, were equivalent in WT and *Ifnlr1^–/–^* lungs on days 2 and 5 p.i. (Supplemental Figure 1C). These data indicate that, in our model system, IFN-λ signaling is not required for the infiltration or neutrophil elastase function of neutrophils following SARS-CoV-2. Furthermore, the enhanced morbidity observed in *Ifnlr1^–/–^* mice is not due to changes in neutrophil function in the absence of IFN-λ signaling.

### IFN-λ signaling regulates CD8 T cell immunity against SARS-CoV-2.

We and others have demonstrated the importance of IFN-λ signaling in regulating expression of the immunosuppressive cytokine IL-10 (22, 47). In addition, expression of the immunosuppressive cytokine *Il10* was increased in the lungs of *Ifnlr1^–/–^* on day 5 p.i. (Figure 3A). Given the role of IL-10 in regulating recruitment and function of immune cells (48), we predicted that endogenous IFN-λ may function to regulate cell-mediated antiviral responses against SARS-CoV-2.

Previous work from our group and others has delineated multiple roles for IFN-λ in the regulation of immune cell responses during viral infection (5, 7). For example, during influenza virus infection, IFN-λ modulates DC, CD8^+^ T cell, and B cell responses (22, 23, 43, 44). Histological analysis revealed no significant changes in overall CD45^+^ cells in WT versus *Ifnlr1^–/–^* lung sections on days 2 or 5 p.i. (Supplemental Figure 1A). However, further pathway analysis of whole lung transcriptomic data indicated significant gene expression changes in *Ifnlr1^–/–^* for pathways associated with immune cell function and recruitment (Supplemental Figure 1E). These data suggest that IFN-λ signaling may also function to regulate cell-mediated immunity of specific populations during SARS-CoV-2 infection that may contribute to outcomes of infection.

Since we observed increases in *Cxcl10* on day 3 p.i. and increased *Il10* on day 5 p.i., we performed flow cytometry on day 4 p.i. to begin to gain an understanding of the potential role of IFN-λ in regulating the accumulation of immune cell subsets (Supplemental Figure 2). We observed similar frequencies (Supplemental Figure 3A) and numbers (Figure 3C) of alveolar macrophages, neutrophils, eosinophils, inflammatory DCs (iDC), plasmacytoid DCs (pDC), NK1.1^+^ cells, B cells, and CD4 T cells in the 2 genotypes. However, the total number of CD8 T cells was significantly increased in *Ifnlr1^–/–^* mice compared with WT (Figure 3C). Strikingly, we observed a significant increase in both the frequency (Supplemental Figure 3A) and number (Figure 3D) of CD103^+^ DC in mice lacking IFN-λ signaling compared with WT. CD103^+^ DC have been shown to express robust levels of IFN-λ receptor, and the activation and migration of CD103^+^ DC to dLN is required for activation of virus-specific CD8 T cells (49, 50). However, day 4 p.i. was too early to detect SARS-CoV-2–specific CD8 T cells in the lungs by tetramer staining (data not shown). Therefore, we performed flow cytometric analysis on day 8 p.i. to evaluate immune cell populations at an effector time point to assess SARS-CoV-2–specific CD8 T cell responses.

**Figure 4:**
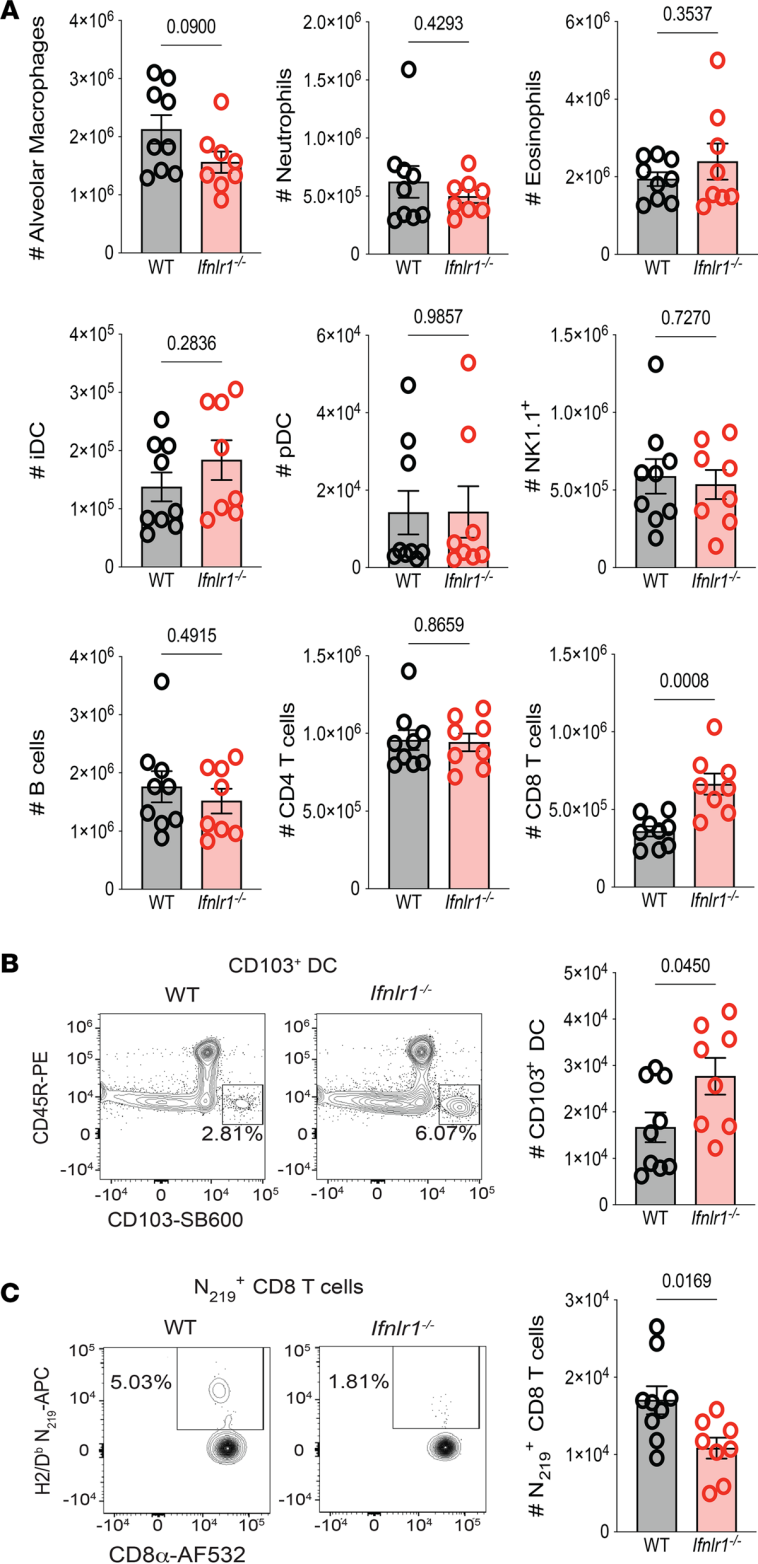
IFN-λ signaling is required for the generation of SARS-CoV-2–specific CD8 T cells in the lung following infection. WT and *Ifnlr1^–/–^* mice were infected intranasally with 1 × 10^5^ TCID_50_ of SARS-CoV-2 MA10. On day 8 p.i., lungs were harvested and numbers of specific immune cell (CD45^+^) populations were determined by flow cytometry. (**A**) Total numbers of alveolar macrophages, neutrophils, eosinophils, CD11c^+^CD11b^+^MHCII^+^ cells (iDC), pDCs, NK cells (NK1.1+), B cells, CD4 T cells, and CD8 T cells in the lungs were determined by flow cytometry. (**B**) Representative flow plots displaying the frequency and graphs quantifying numbers of CD103^+^ DCs of WT and *Ifnlr1^–/–^* mice at day 8 following SARS-CoV-2 MA10 infection. (**C**) Representative flow plots displaying the frequency and graphs quantifying numbers of WT and *Ifnlr1^–/–^* mice at day 8 following SARS-CoV-2 MA10 infection. Data from 2 independent experiments pooled with data representing mean ± SEM. *n* = 8–9 mice/group. Statistical significance was determined by unpaired *t* test. Frequencies on representative flow plots represent the percentage of parent gate (directly upstream of gate named in figure) with gating strategy shown in Supplemental Figure 2.

Similar to day 4 p.i., we observed a significant increase in total CD8 T cells in the lungs of *Ifnlr1^–/–^* compared with WT on day 8 p.i. (Figure 4A), suggesting a potential contribution for IFN-λ signaling to regulate bystander activation of CD8 T cells early following SARS-CoV-2 (51–53). We also observed a significant increase in the frequency and number of CD103^+^ DC in the lungs of *Ifnlr1^–/–^* compared with WT on day 8 p.i. (Supplemental Figure 3B and Figure 4B). In contrast, SARS-CoV-2 N_219_-specific CD8 T cells, which recognize the main immunodominant SARS-CoV-2 epitope in C57BL/6J mice (54), were significantly reduced in the absence of IFN-λ signaling (Supplemental Figure 3B and Figure 4C).

### IFN-λ signaling promotes DC function to support CD8 T cell immunity against SARS-CoV-2.

**Figure 5:**
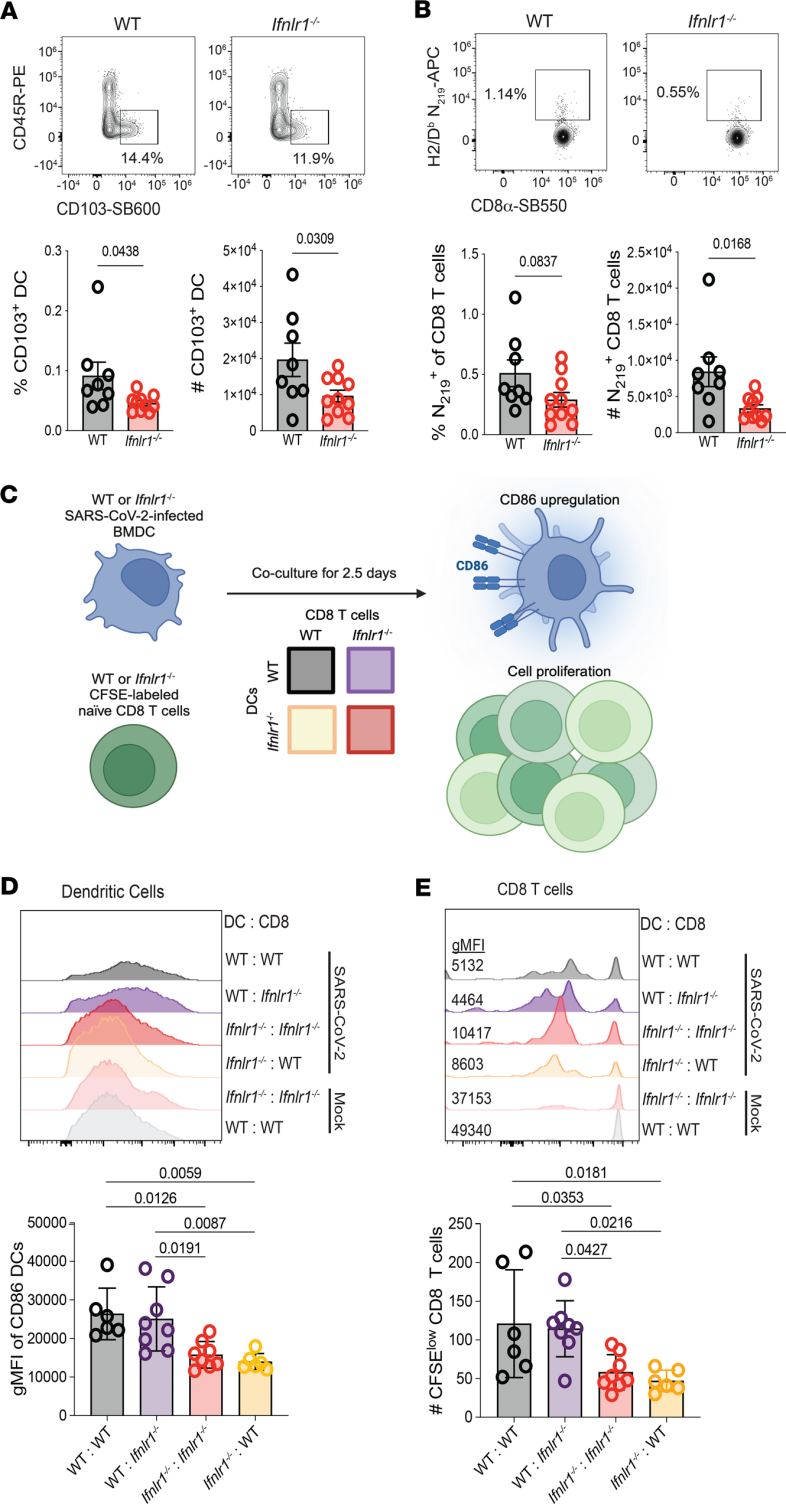
IFN-λ signaling in DC is necessary for CD103^+^ DC and N219^+^ CD8 T cell responses in dLN and CD8 T cell proliferation during SARS-CoV-2 MA10 infection. WT and *Ifnlr1^–/–^* mice were administered 1 × 10^4^ TCID_50_ of SARS-CoV-2 MA10 and, on day 4 p.i., dLN were harvested. (**A** and **B**) The number and frequency of CD103^+^ DCs (**A**) and N_219_-specific CD8 T cells (**B**) were assessed by flow cytometry. Data from 2 independent experiments pooled with data representing mean ± SEM. *n* = 8–11 mice/group. Statistical significance was determined by unpaired *t* test. Frequencies on representative flow plots represent the percentage of parent gate (directly upstream of gate named in figure). (**C**) BMDC generated from WT and *Ifnlr1^–/–^* mice were infected with SARS-CoV-2 and cocultured for 2.5 days with WT or *Ifnlr1^–/–^* CFSE-labeled CD8 T cells purified from spleens of mice. (**D** and **E**) After 2.5 days, CD86 expression on BMDC (**D**) and CD8 T cell proliferation as measured by CFSE dilution (**E**) was quantified by flow cytometry. Statistical significance was calculated by 1-way ANOVA followed by Tukey’s multiple-comparison test. Data from 2 individual experiments pooled with data representing mean ± SEM. *n* = 6–8 mice/group with each data point representing BMDC harvested from an individual mouse.

Since CD103^+^ DC are critical for migration to dLN for activation of virus-specific CD8 T cells, their accumulation in the lungs on day 4 and 8 p.i. suggested an inability of these cells to migrate and accumulate in dLN for CD8 T cell activation in the absence of IFN-λ signaling. Indeed, CD103^+^ DC (Figure 5A) and N_219_-specific CD8 T cell populations (Figure 5B) in dLN on day 4 p.i. were significantly reduced in *Ifnlr1^–/–^* compared with WT. Together, these data indicate that IFN-λ signaling promotes DC migration to dLN, to support activation of a robust CD8 T cell response during SARS-CoV-2 infection.

To directly determine whether IFN-λ signaling in DC or CD8 T cells is responsible for the reduction in SARS-CoV-2–specific CD8 T cell responses in dLN on day 4 p.i. and lungs on day 8 p.i., we performed a coculture experiment where we incubated WT or *Ifnlr1^–/–^* BM-derived DC (BMDC) infected with SARS-CoV-2 with naive, CFSE-labeled WT or *Ifnlr1^–/–^* CD8 T cells enriched from the spleens of mice (Figure 5C). We observed that infection of WT BMDC led to an upregulation of CD86 compared with mock BDMC, and this upregulation upon infection was significantly reduced in *Ifnlr1^–/–^* (Figure 5D). This alteration in CD86 upregulation was dependent solely on the expression of the IFN-λ receptor in BMDC and was independent of the genotype of CD8 T cells. As anticipated based on our in vivo data, coculture of infected WT BMDC with WT CD8 T cells led to activation and division of CD8 T cells as measured by CFSE dilution (Figure 5E) that was significantly reduced during coculture of *Ifnlr1^–/–^* BMDC with *Ifnlr1^–/–^* CD8 T cells (Figure 5E). Strikingly, *Ifnlr1^–/–^* CD8 T cell were able to proliferate to equivalent levels to WT when incubated with WT BMDC (Figure 5E), and proliferation of WT CD8 T cells was significantly reduced when incubated with *Ifnlr1^–/–^* BMDC (Figure 5E). These experiments demonstrate that IFN-λ signaling specifically in DC, and not CD8 T cells, is required for the activation of SARS-CoV-2–specific CD8 T cell responses.

### Control of CD8 T cell immunity during SARS-CoV-2 infection is independent of type I IFN signaling.

**Figure 6:**
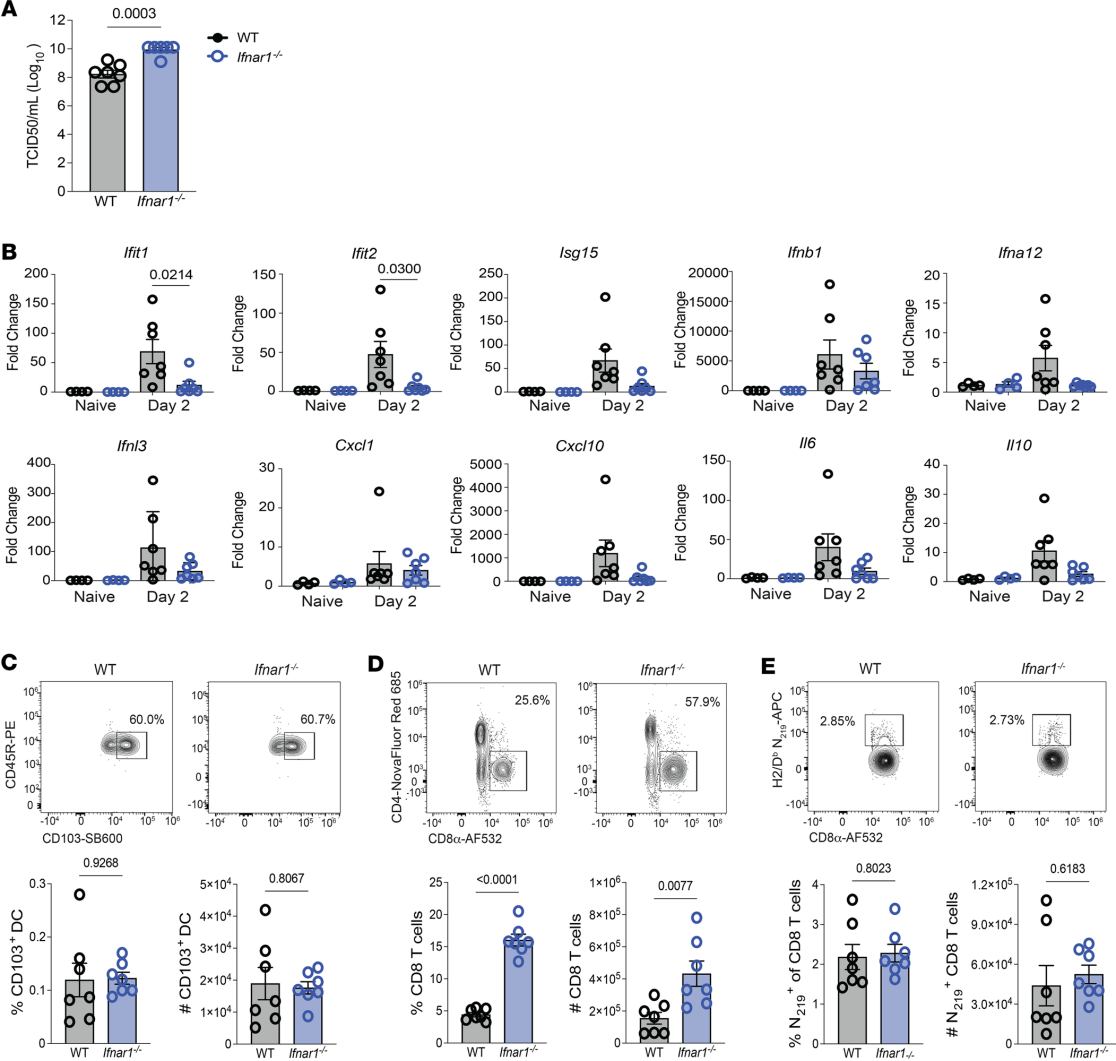
Type I IFN signaling does not affect the generation of SARS-CoV-2 N_219_-specific CD8 T cells in the lungs SARS-CoV-2 MA10 infection. WT and *Ifnar1^–/–^* mice were infected intranasally with 1 × 10^5^ TCID_50_ of SARS-CoV-2 MA10. (**A**) At day 2 p.i., lungs were harvested to quantify virus via TCID_50_. Two independent experiments pooled with data representing mean ± SEM. *n* = 6–7 mice/group. Statistical significance was determined by unpaired *t* test. (**B**) At day 2 p.i., lungs were harvested. Relative expression of *Ifit1*, *Ifit2*, *Isg15*, *Ifnb1*, *Ifna12*, *Ifnl3*, *Cxcl1*, *Cxcl10*, *Il6*, and *Il10* compared with the housekeeping gene *Chmp2a* was determined by qPCR. Statistical significance was calculated by 1-way ANOVA followed by Tukey’s multiple-comparison test. Two independent experiments pooled with data representing mean ± SEM. *n* = 4–7 mice/group. (**C**–**E**) At day 8 p.i., lungs were harvested and the frequency and number of CD103^+^ DCs (**C**), the frequency and number of CD8 T cells (**D**), and the frequency and number of SARS-CoV-2 N_219_-specific CD8 T cells (**E**) were determined by flow cytometry. Data from 2 independent experiments pooled with data representing mean ± SEM. *n* = 7 mice/group. Statistical significance was determined by unpaired *t* test. Frequencies on representative flow plots are percentage of parent gate (directly upstream of gate named in figure).

To determine whether IFN-λ regulates virus-specific SARS-CoV-2 N_219_-specific CD8 T cells via mechanisms distinct from type I IFN, we infected WT and *Ifnar1^–/–^* mice. We have previously reported enhanced susceptibility of *Ifnar1^–/–^* mice to MA10 SARS-CoV-2 infection (25). As expected, and similar to *Ifnlr1^–/–^* mice, the *Ifnar1^–/–^* animals exhibited enhanced susceptibility to SARS-CoV-2 as measured by a nearly 2-log increase in virus titers at day 2 p.i. (Figure 6A). However, assessment of ISGs, IFN, and inflammatory mediators revealed no change in most responses, though significant reductions were observed in *Ifit1* and *Ifit2* expression in *Ifnar1^–/–^* compared with WT on day 2 p.i. (Figure 6B), distinct from the increases observed in *Ifnlr1^–/–^* mice. Total CD8 T cell responses in the lungs were significantly increased in *Ifnar1^–/–^* compared with WT on day 8 p.i. (Figure 6D), similar to *Ifnlr1^–/–^* (Figure 4A). However, in sharp contrast to the phenotype in *Ifnlr1^–/–^* mice (Figure 4, B and C), CD103^+^ DC and N_219_-specific CD8 T cell responses were equivalent between WT and *Ifnar1^–/–^* mice at day 8 p.i. (Figure 6, C and E). While IFN in general regulates the overall magnitude of the CD8 T cell response in the lungs on day 8 p.i., we conclude that IFN-λ signaling uniquely augments the magnitude of SARS-CoV-2–specific CD8 T cell responses during SARS-CoV-2 infection via DC-driven mechanisms that are independent of type I IFN.

## Discussion

Respiratory viral infections, especially those caused by novel pandemic viruses, will continue to be a global health challenge. Understanding how the innate immune response programs long-term protective antiviral immunity will facilitate development of novel prophylactic and therapeutic interventions to combat respiratory viral infections (55–57). Here, we investigated how IFN-λ regulates disease pathogenesis, as well as innate and adaptive immune response to infection using a mouse-adapted SARS-CoV-2 infection. Importantly, this model utilizes endogenously expressed ACE2 for viral entry into cells, allowing us to investigate the contribution of IFN-λ signaling to immunity in a model where infection of cell subsets is more similar to natural infection in humans (33). In agreement with others, we show that IFN-λ signaling confers host protection during SARS-CoV-2 infection, restricting the virus to airways in lung tissue (15–17, 20, 34–36, 58, 59). Importantly, we found that the protective effect of IFN-λ was not solely due to the induction of downstream ISG expression, which was unaltered in *Ifnlr1^–/–^* mice despite delayed virus clearance and enhanced disease. Rather, IFN-λ was required for the induction of transcriptional signatures involved in the cellular immune responses to infection, augmenting SARS-CoV-2–specific CD8 T cell responses and accumulation of CD103^+^ DC in dLN.

Our study revealed that the increased susceptibility to infection as measured by weight loss through day 6 p.i. in *Ifnlr1^–/–^* compared with WT animals could not be attributed solely to differences in the induction of canonical innate immune responses to the virus and virus titers since expression of ISGs, IFN, inflammatory genes, and SARS-CoV-2 titer was only transiently increased in the absence of IFN-λ signaling. This suggests that type I IFN signaling is sufficient to ultimately control virus titers in the absence of type III IFN signaling. Previous in vitro studies have shown an increase in ISGs in the absence of IFN-λ receptor signaling in vitro using epithelial cell lines (16, 17, 34, 35). We extend these observations to in vivo infections, demonstrating that IFN-λ is necessary to regulate early ISG expression and limit viral replication. Given the early increase in SARS-CoV-2 in *Ifnlr1^–/–^* that is ultimately reduced to levels comparable with WT by day 5 p.i. (Figure 1) that is independent of changes we observed in virus-specific CD8 T cell responses, we predict that IFN-λ signaling in multiple compartments (stromal and hematopoietic) contributes to regulating pathogenesis and immunity against SARS-CoV-2. Recent work by Chong et al. utilized mixed BM chimeras to show that IFN-λ signaling primarily from radio-resistant cells restricts viral titer during primary SARS-CoV-2 infection (17). These data support our prediction that unique IFN-λ signaling outcomes in different cell types may regulate distinct facets of viral titer restriction, pathogenesis, and adaptive immune programming.

In our study, we observed a significant increase in total CD8 T cells in the lungs of mice lacking the type I IFN receptor (*Ifnar1^–/–^*) and *Ifnlr1^–/–^* compared with WT mice. However, we observed a significant decrease in the antigen-specific CD8 T cells exclusively found in the lungs of *Ifnlr1^–/–^* mice, and not *Ifnar1^–/–^*, compared with WT. Notably, the observed disparity between total CD8 T cells and virus-specific CD8 T cells in our model may be attributed to antigen-independent activation of CD8 T cells in the absence of IFN-λ signaling (51–53). In the context of SARS-CoV-2 infection, early bystander activation of CD8 T cells characterizes individuals who are asymptomatic or have mild disease symptoms, whereas hospitalized individuals exhibit delayed bystander responses (60), underscoring the pivotal role of bystander CD8 T cell responses in disease outcomes. Studies have shown that type I IFN can directly regulate CD8 T cell dynamics, including bystander activation (61, 62). Meanwhile, IFN-λ acts indirectly on CD8 T cell activation and effector functions through modulation of other immune cells during influenza virus infection (22, 23). However, the exact mechanism underpinning the observed increase in total CD8 T cells in our KO mouse models remains poorly characterized. Future studies to address this knowledge gap will be important for our understanding of IFN dynamics on CD8 T cell immunity.

This study represents one of the first studies to our knowledge to investigate the contribution of type I IFN signaling to development of CD8 T cell responses in a C57BL/6J genetic background utilizing more recently developed mouse-adapted SARS-CoV-2 (33). While type I IFN is an important antiviral effector, the role of type I IFN in regulating CD8 T cell immunity during respiratory virus infection remains controversial and may depend on the infectious agent and the unique inflammatory milieu present in distinct infections (24, 63–65). During influenza virus infection, type I IFN has been shown to promote cytotoxic function of airway CD8 T cells (24). However, type I IFN was dispensable for development of virus-specific CD8 T cell immunity following SARS-CoV infection (65). Likewise, type I IFN is not required for generation of virus-specific CD8 T cells in parainfluenza virus infection of mice (66). To date, other studies have shown a role for type I IFN in generation of CD8 responses during SARS-CoV-2 infections, but these differ from our experimental system because of viral receptor overexpression (63) or the use of a different mouse strain (64), and neither of these previous studies utilized viral epitope–specific tetramer staining to evaluate T cell responses. Given the association of type I IFN–targeting autoantibodies and the association with COVID-19 severity (67, 68), potential preference for IFN-λ rather than type I IFN signaling to promote CD8 T cell immunity may represent a crucial defense mechanism against SARS-CoV-2 infection and COVID-19.

Our findings reveal a unique role for IFN-λ in modulating virus-specific CD8 T cell responses during SARS-CoV-2 infection, as evidenced by equivalent N_219_-specific CD8 T cell responses observed in *Ifnar1^–/–^* mice compared with WT. Our results demonstrate that IFN-λ signaling within DC, and not CD8 T cells, is required for CD8 T cell activation (Figure 5, C–E). This is consistent with a model in which IFN-λ does not act directly on CD8 T cells (22, 69) but instead regulates accumulation and function of CD103^+^ DC in dLN, which are well characterized to effectively cross-present viral antigens to CD8 T cells (70). Our data also suggest a defect in CD103^+^ DC migration, as these cells are retained in the lungs of mice lacking IFN-λ signaling (Figure 3D and Figure 4B) and do not accumulate in dLN (Figure 5A). Importantly, CD8 T cell immunity induced by infection and vaccination provides cross-protection from multiple emerging SARS-CoV-2 strains (28–32). The number of CD8 T cells has been associated as a key correlate of protection, with higher counts associated with milder disease (71–73). Moreover, CD8 T cells targeting structural SARS-CoV-2 proteins are linked to favorable clinical outcomes compared with those targeting nonstructural proteins (72). Understanding strategies to enhance CD8 T cell responses, such as augmentation of IFN-λ signaling, may help to overcome factors that limit protective CD8 T cell immunity.

In this regard, the continued development of IFN-λ as a human therapeutic to limit SARS-CoV-2 disease in individuals with mild COVID-19 may be an effective strategy to engage both innate and adaptive arms of the immune system (18–20). While results from ongoing studies have been mixed, IFN-λ accelerated viral clearance and reduced symptoms when administered within 7 days of symptom onset or a positive test (20). Given our finding that IFN-λ is critical for supporting activation of respiratory virus–specific CD8 T cell immunity (22), it is possible that administration of IFN-λ as a therapeutic during infection, or as a vaccine adjuvant, may bolster this aspect of cell-mediated immunity against SARS-CoV-2. Although recent clinical trials showed no increase in SARS-CoV-2–specific T cell responses on days 7 and 90+ following pegylated IFN-λ (peginterferon lambda) (20), these studies examined only cells isolated from peripheral blood rather than lung tissue. In addition, the trials employed suboptimal peptides for assessing CD8 T cell responses. It is also possible that timing and route of administration in addition to tissue examined could contribute to differing outcomes. In addition, it is possible that the requirement for IFN-λ signaling to lead to full magnitude SARS-CoV-2–specific CD8 T cell responses may not translate to an ability of additional, exogenous IFN-λ to augment SARS-CoV-2–specific CD8 T cell responses. Therefore, the potential for CD8 T cell programming by IFN-λ in relevant human tissues remains an open question in the field that warrants further investigation.

Overall, our study highlights the nonredundant role of IFN-λ signaling for regulating DC accumulation in dLN and subsequent activation of a robust virus-specific CD8 T cell response during SARS-CoV-2 infection. The IFN-λ–driven trafficking and priming was independent of broad changes in anti-viral and ISG responses. Thus, our work significantly extends the known functions of IFN-λ in respiratory virus infections and may inform future therapeutic utilization of IFN-λ, particularly for individuals whose type I IFN responses are subverted, promoting enhanced susceptibility to severe COVID 19.

## Methods

### Sex as a biological variable.

Our infection studies utilized both male and female animals, and we observed similar outcomes for both male and female mice at the age used for infection (6–7 weeks). Pooled male and female data are shown throughout the manuscript.

### Mice.

WT C57BL/6J and *Ifnar1^–/–^* mice were initially purchased from The Jackson Laboratory. These mice and *Ifnlr1*^–/–^ mice are maintained in the vivarium at The Ohio State University. All in vivo infection studies were performed using male and female mice between 6 and 7 weeks of age.

### Virus.

SARS-CoV-2 MA10 was obtained from BEI Resources (NR-55329) (16, 33). Upon receipt, SARS-CoV-2 MA10 was plaque purified on Vero-TMPRSS2 cells (ATCC) (25). Viral stocks were propagated in Vero-TMPRSS2 cells, followed by full genome sequencing to ensure the virus lacked attenuating mutations (74). Titers were determined on viral stock aliquots by tissue culture infectious dose 50 (TCID_50_) assay on Vero E6 cells, as previously described (75).

### Murine infection.

For infection studies, WT, *Ifnlr1*^–/–^, and *Ifnar1^–/–^* mice were anesthetized by isoflurane and challenged intranasally with 50 μL saline for mock controls or 50 μL undiluted SARS-CoV-2 MA10 (6.30 × 10^4^ to 1.13 × 10^5^ TCID_50_/mouse). Mice were monitored for illness and weighed daily. At the designated times after infection, mice were euthanized, and tissues were collected for further analyses described below.

### Histology.

At the designated times after infection, mice were euthanized, and lungs were collected in 10% phosphate buffered formalin for 7 days before being transferred to ethanol. Lung tissue was embedded in paraffin and sectioned at 4 mm. IHC was performed using a Bond Rx autostainer (Leica Biosystems). Histology of lung tissue was performed by HistoWiz Inc. Sequential tissues were stained with H&E, SARS-CoV-2 nucleocapsid, CD45, and Ly6G6C/Gr1 (Supplemental Table 1). Whole slide scanning (40×) was performed in an Aperio AT2 (Leica Biosystems). ImageJ (NIH) was used to quantify the DAB staining of lung tissue. For analyses of each sample, five 10× regions across each tissue section were randomly selected for quantification, and results from these sections were averaged to represent the average pixel intensity.

### RNA isolation and quantitative PCR (qPCR) gene expression.

Following manufacturer protocol, CK14 bead homogenization tubes (Precellys) and TRIzol (Thermo Fisher Scientific) were utilized to homogenize lung tissue from WT and *Ifnlr1*^–/–^ mice in a Precellys 24 homogenizer (Bertin Corp.), and total RNA was isolated (Invitrogen Kit; Ribopure Kit). cDNA was synthesized (Takara, PrimeScript RT Master Mix) and relative gene expression was determined by real-time PCR on a CFX384 Touch Real-Time PCR System (Bio-Rad Laboratories) using primers or primer/probes (Supplemental Table 2) and iTAQ SYBR Green Master Mix (Bio-Rad) or TaqMan Advanced Master Mix (Invitrogen), respectively.

### SARS-CoV-2 N1 quantification.

Copy number qPCR was performed to quantify viral RNA of SARS-CoV-2 (copies/mL) in lung tissue of WT and *Ifnlr1*^–/–^ mice at the designated times p.i. as previously described (76). The pCRII-TOPO vector (Invitrogen) was used to generate a plasmid containing the cDNA coding for the N1 gene of SARS-CoV-2. The N1 plasmid was amplified in competent DH5α *E*. *coli* cells (Invitrogen) and purified (Machery-Nagel, NucleoBond Xtra Midi Kit). N1 RNA transcripts were then synthesized (Invitrogen; Maxiscript kit), purified (Invitrogen; MEGAclear Transcription Clean-Up Kit), and quantified according to manufacturer’s protocol. cDNA was synthesized (Takara, PrimeScript RT Master Mix) from N1 RNA and RNA from whole lung tissue of WT and *Ifnlr1*^–/–^ mice at the designated times p.i. N1 relative gene expression was determined by real-time PCR on a CFX Connect Real-Time PCR System (Bio-Rad) using primers/probe (Supplemental Table 2) and TaqMan Advanced Master Mix (Invitrogen). The N1 RNA standard was used to determine copies/mL viral RNA in each experimental sample.

### RNA-Seq, quality control, data processing, and analysis.

On day 2 and day 5 following SARS-CoV-2 MA10 infection, lungs were isolated from WT and *Ifnlr1*^–/–^ mice along with the lungs of naive WT and *Ifnlr1^–/–^*. Tissue homogenization and total RNA extraction was performed as described above. Library preparation and sequencing was conducted by Aztenta Life Sciences. Briefly, total RNA was quantified by fluorometry (Qubit 2.0 Fluorometer, Thermo Fisher Scientific), and RNA integrity was assessed using a 4200 TapeStation (Agilent). Genomic DNA was removed by treatment with TurboDNase (Thermo Fisher Scientific). Ribosomal RNA was depleted using FastSelect rRNA HMR Kit (Qiagen), and cDNA sequencing libraries were synthesized (NEBNext Ultra II RNA Library Preparation Kit for Illumina), validated (Agilent Tapestation 4200), and quantified via Qubit 2.0 Flurometer (Thermo Fisher Scientific) and qPCR (KAPA Biosystems). cDNA libraries were sequenced (2× 150 bp Paired-End) using an Illumina HiSeq sequencer. Raw RNA-Seq files (fastq) were demultiplexed using Illumina bcl2fastq (v.2.17). Reads were trimmed using cutadapt, and quality scores were assigned via FastQC. Sequencing reads were aligned to the Mus musculus genome buildmm10 using STAR, and gene counts were generated via HTseq using Rosalind (v.3.34.2.2; https://rosalind.bio/). Gene counts were filtered, normalized, and subjected to statistical analysis to determine differential gene expression as previously described (77). Functional analysis of gene expression was performed using Ingenuity Pathway Analysis (IPA).

### TCID_50_ assay.

Lungs of WT, *Ifnlr1*^–/–^, and *Ifnar1^–/–^* mice were harvested in PBS at designated times after infection and homogenized as described above for qPCR analysis, and TCID_50_ was performed on Vero E6 cells using 10-fold serial dilutions in triplicate as previously described (75). Fixed cells were stained for SARS-CoV-2 nucleocapsid antigen, followed by fluorescent secondary antibody detection (Supplemental Table 1). Infected cells were visualized for presence of fluorescence using an EVOS microscope (Invitrogen). TCID_50_ value was calculated using the Reed-Muench method (75).

### Flow cytometry.

On days 4 and 8 following SARS-CoV-2 MA10 infection, mice were euthanized, and lungs were harvested into GentleMACS C tubes (Miltenyi Biotec) containing DMEM with DNase I (Sigma-Aldrich) and type IV collagenase (Worthington Biochemical Corporation). Lungs were processed into single-cell suspensions using GentleMACS (Miltenyi Biotec). Lymph nodes were collected on day 4 p.i. following SARS-CoV-2 MA10 infection and processed into single-cell suspensions using a 100 μm disposable filter (Thermo Fisher Scientific). Single-cell homogenates were blocked in CellBlox (Invitrogen) and stained using eFluor 780 Fixable Viability Dye (Invitrogen) according to the manufacturer’s instructions. Cells were washed and stained with antibodies (Supplemental Table 1) and H-2D^b^ N_219-227_ tetramer (NIH Tetramer Core) for 30 minutes on ice. Cells were fixed with BD FACS lysis buffer for 1 hour at 4°C, resuspended in PBS, and run on the Cytek Aurora with count beads (Invitrogen) added to each sample for quantification. Samples were analyzed using FlowJo Software (version 10.8.1).

### In vitro coculture of DCs and CD8^+^ T cells.

Spleens were isolated from animals, and homogenization was performed as described above. Splenic CD8^+^ T cells were isolated (Miltenyi Biotec; CD8a^+^ T cell Isolation Kit) and CFSE was diluted according to manufacturer’s protocol (Invitrogen; CellTrace CFSE Cell Proliferation Kit). CFSE labeling was performed by resuspending CD8^+^ T cells at 1 × 10^7^ cells/mL in 2.5 μM of CFSE in PBS with 0.1% BSA. The cells were incubated for 20 minutes in the dark at 37°C, and the reaction was quenched with the addition of 5 volumes of RPMI media (Thermo Fisher Scientific) for a 5-minute incubation period. Femur and tibia BM was harvested from WT and *Ifnlr1*^–/–^ mice. BM cells were cultured for 7 days in media supplemented with 20 ng/mL of murine IL-4 (PeproTech) and 20 ng/mL of murine GM-CSF (PeproTech) to differentiate into BMDCs. BMDCs were plated in a 96-well round-bottom plate and infected with SARS-CoV-2 MA10 at a MOI of 0.1 for 2 hours at 37°C. Following infection, BMDCs were washed with 1× PBS (Thermo Fisher Scientific) and resuspended with CFSE-labeled CD8^+^ T cells at a 1:10 ratio of CD8^+^ T cells to DCs. At 36-hours after incubation, the cells were washed and stained with antibodies (Supplemental Table 1). Samples were prepared for flow analysis, as described above.

### Statistics.

Statistical analyses and data visualization were performed using GraphPad Prism Software (version 9.3.1). The specific statistical analyses used are described in the figure legends and include unpaired, 2-tailed t tests, 2-way ANOVA with Šidák’s multiple-multiple comparison test, and 1-way ANOVA with Tukey’s multiple-comparison test. A *P* value less than 0.05 was considered significant. Any comparison between WT and *Ifnlr1^–/–^* or *Ifnar1^–/–^* on a specific day without a comparison noted is not statistically significant. Comparisons between analysis days are not included in presented data.

### Study approval.

All animal experiments were performed following approved IACUC and animal biosafety level 3 (BSL3) protocols at The Ohio State University.

### Data availability.

Individual values for data sets are deposited in the Supporting Data Values file. The RNA-Seq data generated in this study are available via the following accession identifiers on the NCBI database (GEO: GSE253635).

## Author contributions

ADS designed experiments, conducted experiments, analyzed data, and wrote the manuscript. PJD conducted experiments. ADK conducted experiments. NSM conducted experiments. SS conducted experiments. QG assisted in conducting experiments and managed the mouse colony. RTR provided critical reagents. MEL provided critical reagents. AF provided input on study design and analyzed data. JSY provided critical reagents. EAH conceived of the study, designed experiments, performed experiments, analyzed data, and wrote the manuscript. All authors edited and approved the final version of the manuscript.

## Supplementary Material

Supplemental data

Supporting data values

## Version 1 07/08/2024

Electronic publication
