# Supplementary material for: IFN-λ uniquely promotes CD8 T cell immunity against SARS-CoV-2 relative to type I IFN
Source: JCI Insight. 2024 May 21;9(13):e171830. doi: 10.1172/jci.insight.171830 (PMC11383353; doi:10.1172/jci.insight.171830)
Supplement: Supplemental data [file jciinsight-9-171830-s129.pdf]

Solstad et al. Supplementary Figures

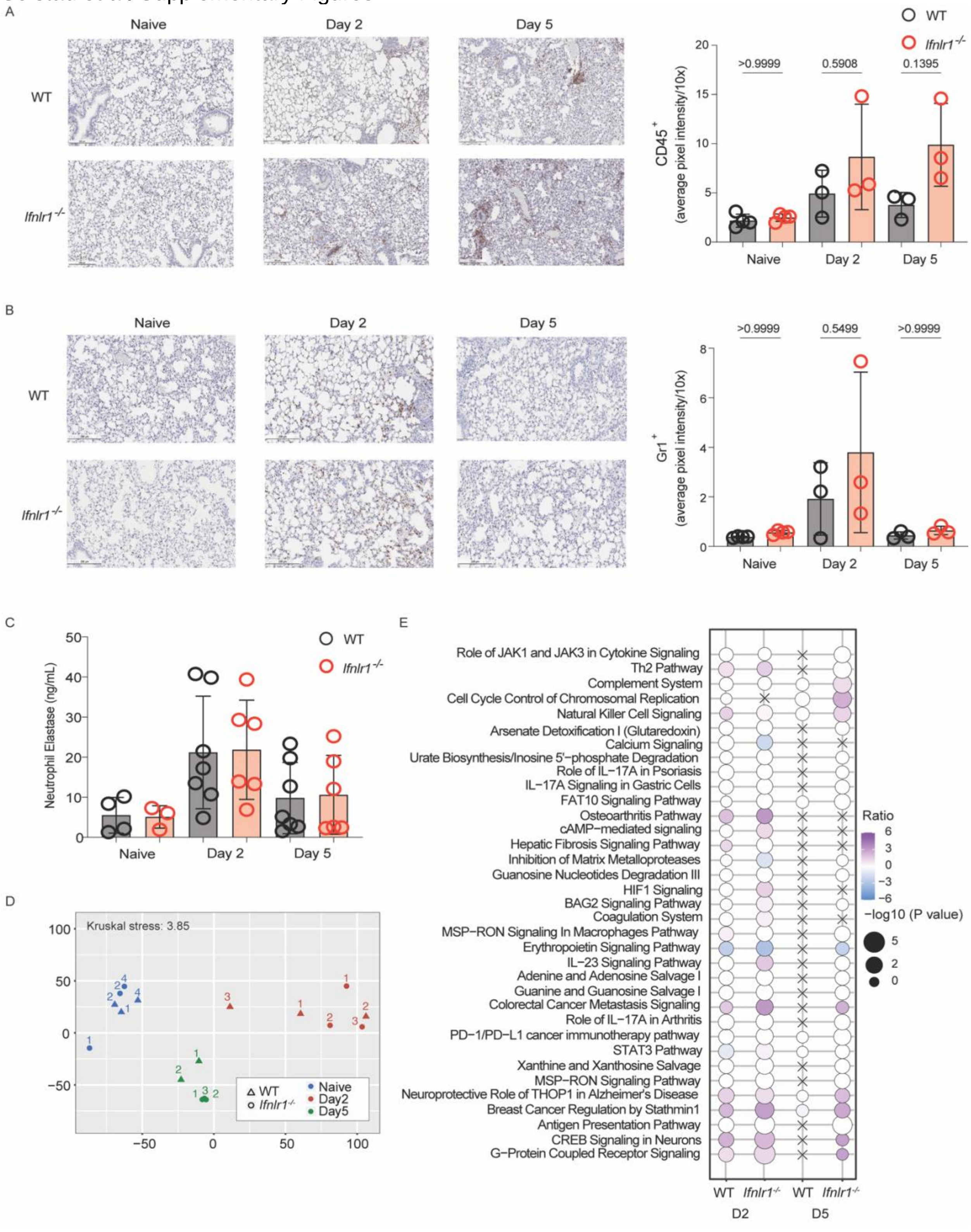

Supplementary Figure 1. IFNλ signaling does not affect lymphocyte or granulocyte infiltration during SARS-CoV-2 MA10 infection. WT and *Ifnlr1*$^{-/-}$ mice were infected intranasally with 10^5 $TCID_{50}$ of SARS-CoV-2 MA10. Lungs were harvested from naïve mice, or on day 2 and 5 post infection. Immunohistochemistry was performed staining for **(A)** CD45.2 and **(B)** Gr1. Staining was quantified via ImageJ by averaging the pixel intensity of ten randomly selected 10X images. Error bars represent +/- SD. Significance was determined using ordinary one-way ANOVA with Tukey's multiple comparisons test. n=3-4 mice/group. **(C)** Amount of neutrophil elastase in whole lung homogenates was determined by ELISA. Data from two independent experiments pooled. n=3-7 mice/group. **(D)** Transcriptional profiling of the pulmonary host response to SARS-CoV2 infection at day 2 and day 5 post infection. Multidimensional scaling (MDS) of the similarities in the global transcriptional profiles of murine lungs infected with SARS-CoV-2. Each data point represents a biological replicate (n=3-4 mice/group). Shape represents genotype and color indicates time-post infection. The low percentage of Kruskal stress (3.85%) suggests a faithful two-dimensional scaling of global transcriptional differences between genotypes and timepoints. **(E)** Ingenuity pathway analysis was used to plot pathways significantly up- or down-regulated in WT or *Ifnlr1*$^{-/-}$ lungs during SARS-CoV-2 infection.

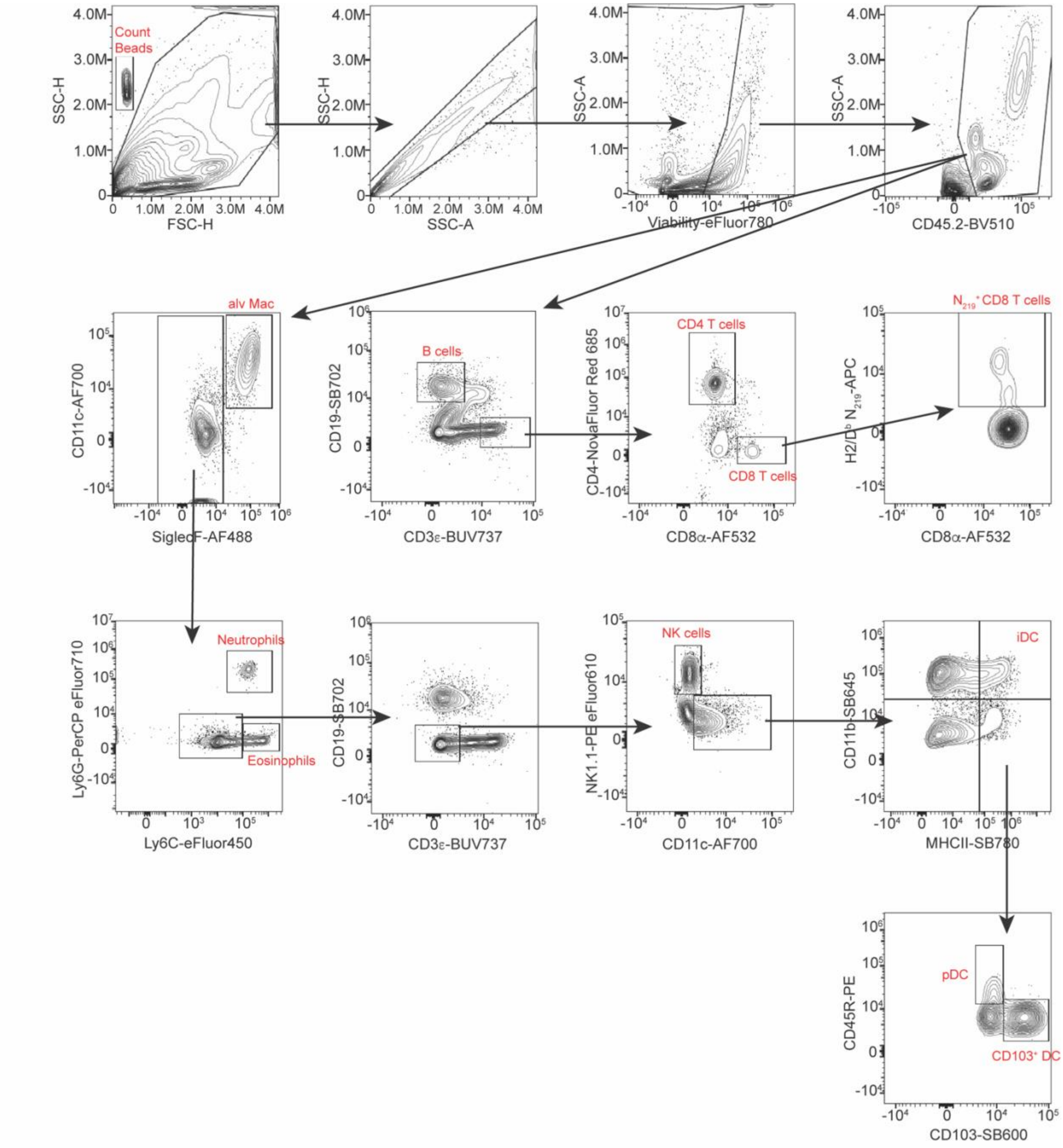

Supplementary Figure 2. Gating strategy for analyses of immune cell populations. Whole lungs and lymph nodes were harvested from WT and *Ifnlr1*$^{-/-}$ mice from mock-infected or at day 4 and 8 post infection. Cells were stained with antibodies to assess alveolar macrophages, B cells, CD4^{+} T cells, CD8^{+} T cells, SARS-CoV-2 $N_{219}$$^{+}$-specific CD8 T cells, neutrophils, eosinophils, NK cells, iDCs, pDCs, and CD103^{+} DCs (CD45.2, CD11c, CD11b, SiglecF, Ly6G, Ly6C, MHCII, NK1.1, CD19, CD3e, CD4, CD45R). CountBright Plus Absolute Counting Beads were utilized to calculate cell number for each immune cell subset.

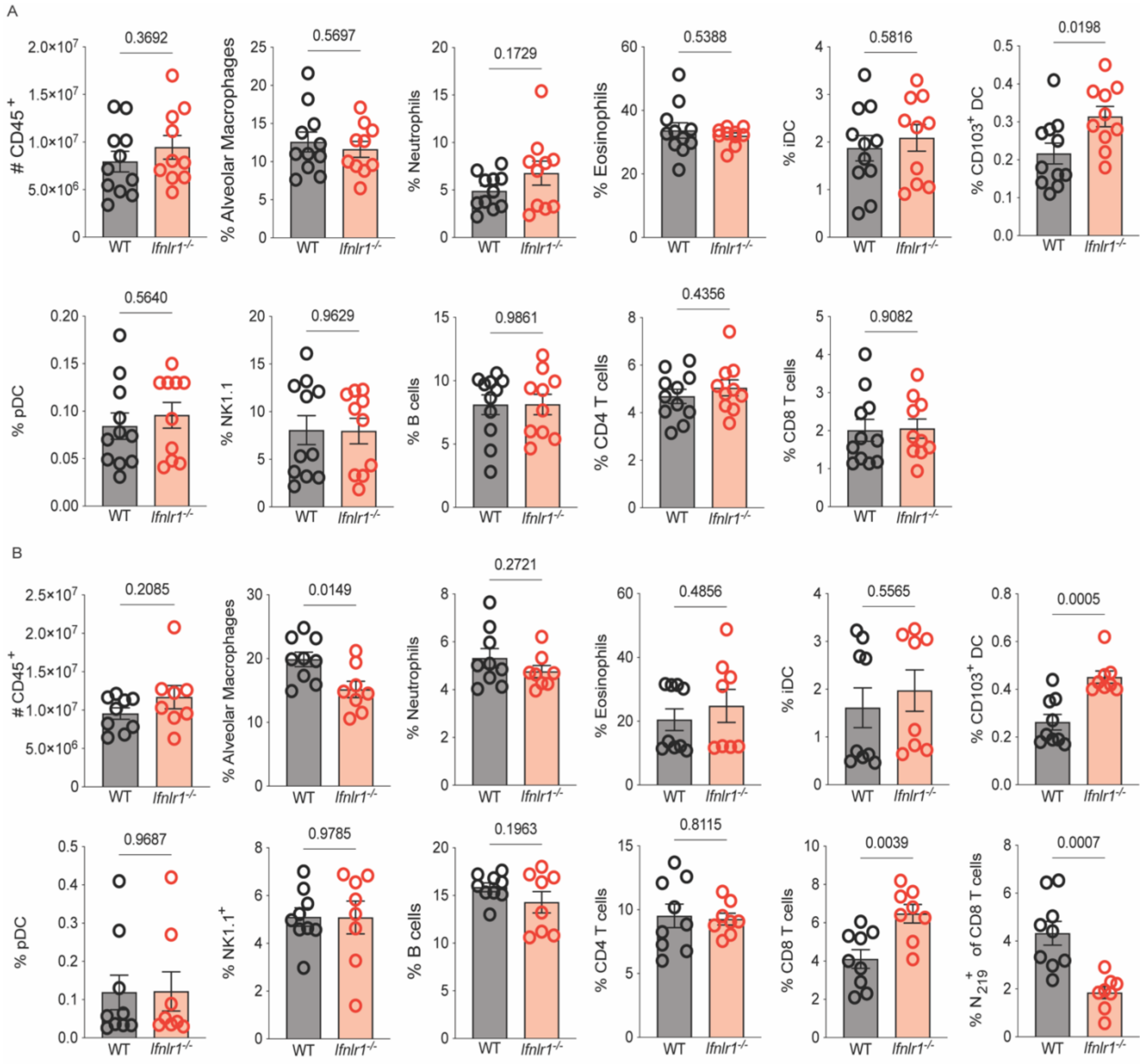

Supplementary Figure 3. IFNλ signaling regulates CD103+ DC and SARS-CoV-2 N_{219}^{+}-specific CD8 T cells in the lungs during SARS-CoV-2 MA10 infection. At day 4 (**A**) and 8 (**B**) post infection**,** lungs were harvested from WT and *Ifnlr1$^{-/-}$* mice infected intranasally with 10^5 $TCID_{50}$ of SARS-CoV-2 MA10. The total numbers of $CD45^+$ cells as well as percentages of alveolar macrophages, neutrophils, eosinophils, iDCs, $CD103^+$ DCs, pDCs, NK cells (NK1.1+), B cells, CD4 T cells, CD8 T cells, and SARS-CoV-2 N_{219}^{+}-specific CD8 T cells were assessed by flow cytometry. Data from two independent experiments pooled with error bars representing SEM. n=8-11 mice/group. Statistical significance was determined by unpaired t-test.

Supplementary Methods Table 1. Antibodies used for flow cytometry, $TCID_{50}$ and IHC.						
**Antigen**	**Fluorophore**	**Clone**	**Isotype**	**Company**	**Catalog Number**	**Lot Number**
Flow Cytometry Antibodies						
CD3e	BUV737	17A2	Rat IgG2b	Invitrogen	367-0032-82	2448221
CD3e	PerCP-Cy5.5	145-2C11	Armenian Hamster IgG	TONBO Biosciences	650031U100	C0031110823653
CD4	NovaFluor Red 685	GK1.5	Rat IgG2b	Invitrogen	M001T02R02	C021465-AT031357
CD8a	AF532	53-6.7	Rat IgG2a	Invitrogen	58-0081-80	2355934
CD8a	SparkBlue 550	53-6.7	Rat IgG2a, k	BioLegend	100780	B390415
CD11b	SB645	M1/70	Rat IgG2b	Invitrogen	64-0112-82	2363794
CD11c	AF700	N418	Armenian Hamster IgG	BioLegend	117320	B377175
CD19	SB702	1D3	Rat IgG2a	Invitrogen	67-0193-82	2383374
CD44	BV785	IM7	Rat IgG2b, k	BioLegend	103059	B307525
CD45.2	BV510	104	Mouse (SJL) IgG2a	BioLegend	109838	B323842
CD45R	PE	RA3-6B2	Rat IgG2a, k	Invitrogen	12-0452-82	2213766
CD69	PE-Cy7	H1.2F3	Armenian Hamster IgG	Invitrogen	25-0691-82	2349859
CD86	PE	A17199A	Rat IgG2a, k	BioLegend	159203	B344080
CD103	SB600	2E7	Armenian Hamster IgG	Invitrogen	63-1031-82	2233917
Ly6G	PerCP-eF710	1A8	Rat IgG2a, k	Invitrogen	46-9668-82	2321313
Ly6C	eF450	HK1.4	Rat IgG2c, k	Invitrogen	48-5932-82	2313096
MHCII	SB780	M5/114.15/2	Rat IgG2b, k	Invitrogen	78-5321-82	2416185
NK1.1	PE-eF610	PK136	Mouse IgG2a, k	Invitrogen	61-5941-82	2297315
SiglecF	AF488	1RNM44N	Rat IgG2a, k	Invitrogen	53-1702-82	2271475
$TCID_{50}$ Antibodies						
SARS-CoV-2 Nucleocapsid	NA	08	Mouse IgG1	Sino Biological	40143-MM08	NA
Mouse IgG	AF488	polyclonal	Goat IgG	Invitrogen	A11029	2486523
IHC Antibodies						
CD45	NA	polyclonal	Rabbit IgG	Abcam	ab10558	NA
Ly6G6C/Gr1	NA	RB6-8C5	Rat IgG2b	Abcam	ab25377	NA
SARS-CoV-2 Nucleocapsid	NA	HL448	Rabbit IgG	GeneTex	GTX635686	NA

Supplementary Methods Table 2. Primers and primer/probe sets used for gene expression assays.			
**Gene**	**Forward**	**Reverse**	**Company**
Chmp2a	5'-AGACGCCAGAGGAACTACTTC-3'	5'-ACCAGGTCTTTTGCCATGATTC-3'	IDT
Cxcl10	5'-CCAAGTGCTGCCGTCATTTTC-3'	5'-GGCTCGCAGGGATGATTTCAA-3'	IDT
Ifit1	5'-CTGAGATGTCACTTCACATGGAA-3'	5'-GTGCATCCCCAATGGGTTCT-3'	IDT
Ifit2	5'-CTGGGGAAACTATGCTTGGGT-3'	5'-ACTCTCTCGTTTTGGTTCTTGG-3'	IDT
Il6	5'-CCAAGAGGTGAGTGCTTCCC-3'	5'-CTGTTGTTCAGACTCTCTCCCT-3'	IDT
Isg15	5'-GGTGTCCGTGACTAACTCCAT-3'	5'-TGGAAAGGGTAAGACCGTCCT-3'	IDT
**Gene- Primer/Probe Set**		**Catalog Number**	
nCoV-N1 Forward		10006830	IDT
nCoV-N1 Reverse		10006831	IDT
nCoV-N1 probe		10006832	IDT
Gapdh		Mm9999915_g1	ThermoFisher
Ifna12		Mm00616656_s1	ThermoFisher
Ifnb1		Mm00439552_s1	ThermoFisher
Ifnl3		Mm00663660_g1	ThermoFisher
Il10		Mm01288386_m1	ThermoFisher
